# Biosorption of Pb (II) from aqueous solution by extracellular polymeric substances extracted from *Klebsiella* sp. J1: Adsorption behavior and mechanism assessment

**DOI:** 10.1038/srep31575

**Published:** 2016-08-12

**Authors:** Wei Wei, Qilin Wang, Ang Li, Jixian Yang, Fang Ma, Shanshan Pi, Dan Wu

**Affiliations:** 1 School of Municipal and Environmental Engineering, Harbin Institute of Technology, Harbin 150090, People’s Republic of China; 2 State Key Laboratory of Urban Water Resource and Environment, Harbin Institute of Technology, Harbin 150090, People’s Republic of China; 3 Advanced Water Management Centre, The University of Queensland, St Lucia, Queensland 4072, Australia

## Abstract

The adsorption performance and mechanism of extracellular polymeric substances (EPS) extracted from *Klebsiella* sp. J1 for soluble Pb (II) were investigated. The maximum biosorption capacity of EPS for Pb (II) was found to be 99.5 mg g^−1^ at pH 6.0 and EPS concentration of 0.2 g/L. The data for adsorption process satisfactorily fitted to both Langmuir isotherm and pseudo-second order kinetic model. The mean free energy *E* and activation energy *Ea* were determined at 8.22– 8.98 kJ mol^−1^ and 42.46 kJ mol^−1^, respectively. The liquid-film diffusion step might be the rate-limiting step. The thermodynamic parameters (*ΔG*^*o*^, *ΔH*^o^ and *ΔS*^*o*^) revealed that the adsorption process was spontaneous and exothermic under natural conditions. The interactions between EPS system and Pb (II) ions were investigated by qualitative analysis methods (i.e Zeta potential, FT-IR and EDAX). Based on the strong experimental evidence from the mass balance of the related elements participating in the sorption process, an ion exchange process was identified quantitatively as the major mechanism responsible for Pb (II) adsorption by EPS. Molar equivalents of both K^+^ and Mg^2+^ could be exchanged with Pb^2+^ molar equivalents in the process and the contribution rate of ion exchange to adsorption accounted for 85.72% (Δmequiv = −0.000541).

Heavy metal contamination of water bodies presents a severe hazard to public health and environment owing to their accumulation in the food chain as non- biodegradable pollutants and persistence in nature^1^. Lead is used as an industrial raw material for electric battery manufacturing, plating, mining, and tanneries^2^. Due to its acute and chronic toxic effects in animal and human health, Environmental Protection Agency (EPA) standard for lead in drinking water and wastewater is 0.05 and 0.5 mg L^−1^, respectively^3^. However, concentrations of lead ions from 20 to 400 mg L^−1^ have been reported in wastewaters from some of the preceding industrial sources, which is much higher than the prescribed limit by EPA^4^. Therefore, its concentration must be carefully controlled and monitored to levels complying with environmental regulations.

To date, numerous methods (e.g. ion exchange, chemical precipitation, membrane technologies, electrochemical treatment, and evaporation) have been developed for removing heavy metals from contaminated water^5^. However, most of these methods suffer from some drawbacks, such as energy consumption, high reagent or the disposal of the residual floc residues that cause secondary pollution, and especially are not suitable for low concentrations of dissolved metal(s) ranging between 1 and 50 mg L^−1^ 6,7. Biosorption has been portrayed as a promising and eco-safety technique, which allows the levels of hazardous metal pollution to meet the requirement of relevant environmental regulations for various water bodies^8^. Recent research focuses on developing novel, effective, low-energy consumption and easy-to-operate biosorbents. Living and dead cells of microorganisms and waste biomaterials from other industries or by-products can be utilized in this manner^9^. Although freely cells have better contact with the metal ions, the biomass suspension is normally not used in large-scale biosorption processes. This is because the microbial biomass often requires immobilization to enhance its stability and mechanical strength, which increases the complexity of the operation^10^. Although several waste biomaterials show good adsorption efficacy, they require extremely high process temperature levels, which results in extra energy consumption^11^,^12^. Thus, different types of biomasses as alternative biosorbents have been proposed to remove heavy metals.

Extracellular polymeric substances (EPS), derived from metabolism of microbial cells in the culture medium. Numerous studies have now shown that carbohydrates and proteins are found to be the major components of EPS and various metallic elements (e.g. Fe, Mg, K, Mn) are present in the EPS as well. EPS was considered as an organic product with mineral cations bound^13^,^14^. EPS possess an abundance of negatively charged functional groups, which can react with heavy metals as ligands^15^. The properties of EPS are providing unprecedented opportunities for the removal of hazardous metal in highly efficient, non-toxicity, low-energy consumption and easy-to-operate approaches, and various EPS have been exploited for this purpose. According to the previous studies, EPS extracted from different types of bacteria such as *Pseudomonas* sp., *Rhodococcus* sp., *Paenibacillus* sp., *Shewanella* sp., *Desulfovibrio* sp., have presented considerable potential in heavy metal detoxification^16^,^17^,^18^,^19^,^20^. However, biosorption efficiency and parameters were of key interest in those studies. The biosorption mechanisms (e.g. complexation, ion exchange, charge neutralization and chelation) of EPS have not been systematically studied in a quantitative way and therefore there were no conclusive conclusions about the main mechanism of heavy metal biosorption in the previous studies. Although biosorption parameters are required for practical application, identification of the major adsorption mechanism is also of great importance, which would facilitate the selection of biosorption materials.

Our previous studies showed that EPS extracted from *Klebsiella* sp. J1, which existed widely in natural water and grain, had emerged as a promising biosorbent for the removal of low concentrations hazardous metals (e.g. Cd^2+^, Cu^2+^, Zn^2+^) in both single and binary metals wastewater systems^21^,^22^. It was different from other EPS extracted from different microbial species (e.g. *Paenibacillus polymyxa*, *Rhodococcus erythropolis*) due to its higher protein content, which was nearly 1.5 times as much as the content of the polysaccharide. In addition, *Klebsiella* sp. J1 cells were cultured and incubated in liquid medium containing some metallic elements (e.g. Mg, K). Thus, a part of mineral cations were bound to the EPS organic matter. However, our works in the past focused on synergetic effects of anionic polyacrylamide and EPS and competitive adsorption of heavy metals rather than adsorption mechanism assessment of EPS. The adsorption mechanism assessment would promote exploitation of highly efficient and cost-effective biosorbent and develop biosorption process towards a more profitable and economically viable process^23^.

Therefore, this study aimed to investigate the utility of EPS extracted from *Klebsiella* sp. J1 to remove Pb (II) in low-level aqueous systems and systematically study the adsorption mechanisms of EPS with both qualitative and quantitative analysis approaches. Attempts have been made to understand the factors responsible for adsorption of Pb (II) to EPS. Isotherm, kinetic and thermodynamic parameters were also evaluated to describe the adsorption mechanism changed into processes. The interactions between biosorbent system and Pb (II) ions were investigated by qualitative analysis methods (i.e. Zeta potential, FT-IR and EDAX). According to mass balance of elements in liquid and solid phase before and after Pb (II) removals, the major mechanism was identified quantitatively and its contribution rate to adsorption was calculated.

## Results and Discussion

### Removal of Pb (II) from synthetic solutions

#### Effect of the mass of EPS

**Figure 1:**
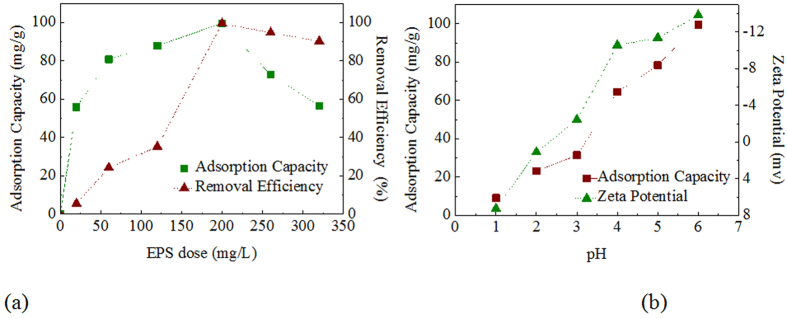
Effect of (**a**) EPS of *Klebsiella* sp. J1 dose, (**b**) pH on the biosorption of Pb (II).

The effect of EPS extracted from *Klebsiella* sp. J1 dosage on the retention of Pb (II) was studied using different mass in the range of 20–320 mg L^−1^ to treat 100 mL of 20 mg L^−1^ Pb (II) solution and the results were presented in Fig. 1(a). The removal efficiency of Pb (II) increased with increasing dose of EPS and reached a maximum (99.47%) at around 200 mg L^−1^ EPS. However, the removal efficiency decreased when the EPS concentration exceeded the optimal level. This result indicated a greater or less dose would be unfavorable for the removal of Pb (II) from synthetic solutions. Therefore, the optimum EPS dose was taken as 200 mg L^−1^ and this was used for all further studies. The positive correlation within a certain range between biosorbent dose and retention of Pb (II) can be related to the increasing available binding sites. However, if the mass of EPS exceeded the optimal level, a partial of EPS molecules would aggregate and form bridging bonds, which resulted in a decrease in effective surface area and sorption sites for Pb (II), leaving metal ions free^18^. It should be noted that although the minimum concentration of lead was 20 mg L^−1^ tested, the lower initial Pb (II) concentrations (i.e. 5, 10 and 15 mg L^−1^) also affected the amount of adsorbed lead and the result was presented in Fig. S1. The result showed that the sorption capacity of Pb (II) increased with increasing initial Pb (II) concentration, which was due to higher availability of lead ions for the biosorption. Moreover, higher initial Pb (II) concentration provided increased driving force to overcome all mass transfer resistance of lead ions between the aqueous and solid phase^10^. This resulted in higher probability of collision between lead ions and EPS. This also leaded to higher metal Pb (II) uptake.

#### Effect of pH

pH is an important controlling factor for both solution chemistry of metallic ions and functional groups characteristics of biosorbent. At the pH values of greater than 7, Pb (II) ions became precipitate as Pb(OH)_2_ due to increasing concentration of OH^−^ ions in the solution^10^. For this reason, the effect of the hydrogen ion concentration was conducted in the pH range of 1.0–6.0. Under these circumstances, Pb^2+^ and Pb(OH)^+^ were the dominant species^2^. As shown in Fig. 1(b), the adsorption capacity of EPS (EPS at 200 mg L^−1^) of *Klebsiella* sp. J1 for Pb (II) (concentration at 20 mg L^−1^) was positively correlated with elevated pH and reached the maximum value (99.47 mg g^−1^) at pH 6.0. It should be noted that the wastewater after its treatment was able to reach a level (i.e. 0.1 mg L^−1^) that satisfied the EPA standard for wastewater discharge. The zeta potential of EPS was measured as positive at the pH between 1.0 and 2.0 and the overall surface of EPS was negatively charged at the pH between 3.0 and 6.0. The minimum negative zeta potential value (zP = −13.82 mV) was observed at pH 6.0, which corresponded to the maximum adsorption capacity of EPS to Pb (II).

At low pH values, EPS surface was completely covered with hydrogen ions and the Pb (II) ions could not compete with them for adsorption sites^2^. At the same time, the activity of functional groups of EPS (e.g., hydroxyl and carboxyl) was affected by pH and this could be explained by the surface charge change on the functional groups of EPS^24^. At low pH values (pH 1–2), functional groups of EPS gained protons and the zeta potential of EPS was measured as positive. Increased positive charge (protons) density on functional groups of EPS at low pH values restricted the approach of positively charged Pb (II) as a result of repulsive force. However, the competition from the hydrogen ions decreased with the increasing pH, and more functional groups of EPS became negatively charged and electrostatic repulsion decreased, with subsequent attraction of metal cations and adsorption on the EPS surface. A similar theory was proposed in several earlier works for metal adsorption on different adsorbents^2^,^18^,^24^.

A comparative study of the biosorption capacity for Pb (II) under similar conditions was carried out with other reported biosorbents (Table 1). The data revealed that the removal capacity of EPS extracted from *Klebsiella* sp. J1 for Pb (II) was higher than the majority of other biosorbents that have been mentioned except for the functionalized *Saccharomyces cerevisiae* cell. However, it required immobilization to enhance its stability and mechanical strength, which increased the complexity of the operation^25^. Therefore, it could be noteworthy that EPS extracted from *Klebsiella* sp. J1 was a potential biosorbent for the removal of solute Pb (II) ions.

#### Biosorption isotherms

**Figure 2:**
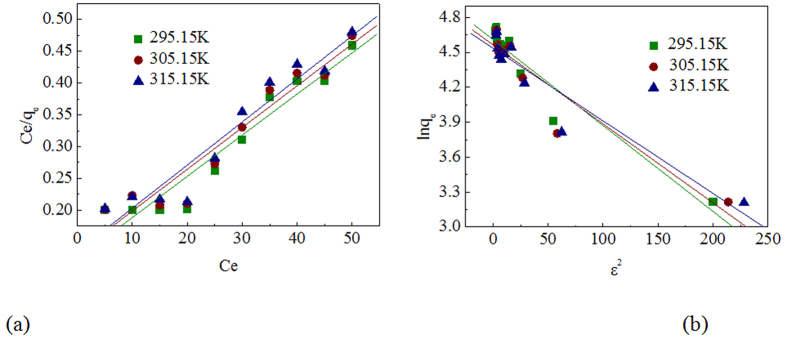
Isotherm plots for the adsorption of Pb (II) onto EPS of *Klebsiella* sp. J1. (**a**) Langmuir isotherm, (**b**) Dubinin-Radushkevich isotherm.

Equilibrium isotherms are important in the design of adsorption systems, which indicate surface properties and affinity of the biosorbent and how the metal ions are distributed between the biosorbent and liquid phases at equilibrium. Several empirical models such as Langmuir, Freundlich and Dubinin-Radushkevich models were used to characterize the adsorption equilibrium and the biosorption isotherm plots for the Pb (II) adsorption onto EPS of *Klebsiella* sp. J1 were presented in Fig. 2. The plots for the Freundlich isotherm model were not shown because the coefficients of determination for this model were low (*R*^2^ < 0.8) for the Pb (II) biosorption at the studied temperatures of 295.15–315.15 K. The parameters of three models for the biosorption of Pb (II) onto EPS were listed in Table 2.

It was observed that the adsorption behavior of Pb (II) ion onto EPS was characteristic to the Langmuir model (*R*^2^ > 0.9) compared to the Freundlich model (*R*^2^ < 0.8) under the concentration range studied. The Langmuir isotherm model suggested a monolayer sorption on homogeneous surface without interaction between adsorbed Pb (II) ions. Once the monolayer coverage was achieved, the sorption was no longer influenced significantly by the solute transport and a strong affinity of the solute on EPS at low concentration could be shown^2^. In addition, the Langmuir constants *q*_*m*_ and *b* exhibited decrease with a rise in temperature, indicating the exothermic nature of the process. The essential feature of the Langmuir isotherm can be expressed in terms of a dimensionless factor (separation factor *R*_*L*_), which is defined by equation (1):


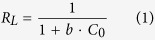


where *C*_*0*_ (mg L^−1^) is the initial concentration of Pb (II) and *b* (L mg^−1^) is the Langmuir isotherm constant related to the heat of adsorption. The value of R_L_ indicates the adsorption system to be either unfavorable (*R*_*L*_ > 1), linear (*R*_*L*_ = 1), irreversible (*R*_*L*_ = 0) or favorable (0 < *R*_*L*_ < 1)^26^. The values of R_L_ calculated at different temperatures were given in Table 2 which confirmed a highly favorable uptake of Pb (II) in the adsorption process. The Dubinin-Radushkevich isotherm model was applied to distinguish the adsorption nature as physical or chemical. A value of *E* between 8 and 16 kJ mol^−1^ corresponds to adsorption by ion exchange, while a value of E lower than 8 kJ mol^−1^ corresponds to physical adsorption^24^. Accordingly, the numerical value of the mean free energy *E* at different temperatures (8.22 kJ mol^−1^, 8.57 kJ mol^−1^, 8.98 kJ mol^−1^) might correspond to a ion-exchange mechanism. Similar results were also obtained in the research of Cabuk *et al*.^10^.

#### Kinetic studies

**Figure 3:**
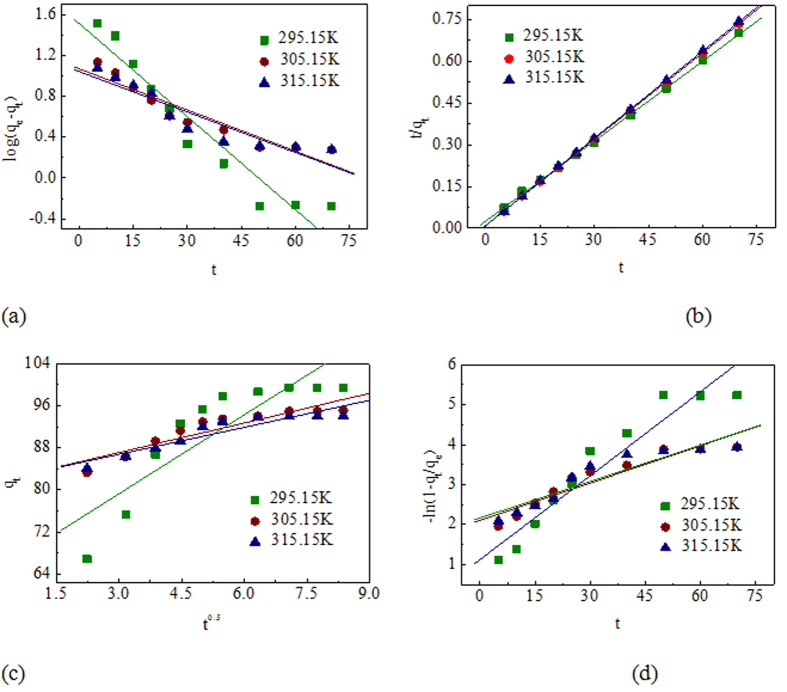
Kinetic plots for the adsorption of Pb (II) onto EPS of *Klebsiella* sp. J1. (**a**) Pseudo-first order model, (**b**) pseudo-second order model, (**c**) intra-particle diffusion model, (**d**) liquid-film diffusion model.

Kinetic analysis is required to understand the rate of biosorption and the rate-limiting step of the transport mechanism. Pb (II) adsorption rate depended on residence time of the metal ion at the solid-solution interface and diffusion process. 100 mL of 20 mg L^−1^ Pb (II) solution with 320 mg L^−1^ EPS were agitated on a jar test apparatus. Samples were collected at definite time intervals (5 min to 70 min) and filtered. Residual metal concentration in the filtrates was analyzed by Inductively coupled plasma optical emission spectrometry and adsorption capacity *qt* (mg g^−1^) at any time *t* (h) were calculated. The pseudo-first order and pseudo-second order models were employed to evaluate the kinetic mechanism process and the results were shown in Fig. 3(a,b), respectively. The kinetic parameters for these two models were calculated and tabulated in Table 3.

The fitting of kinetic data in pseudo-second order expression showed excellent linearity with high correlation coefficient (*R*^2^ > 0.99) over the temperature range studied. The calculated value of q_e_ from the first-order kinetics model was observed to be dramatically lower than the experimental value. Therefore, an analysis of the data implied that the adsorption of Pb (II) onto EPS of *Klebsiella* sp. J1 followed pseudo-second order kinetics. Some very recent investigations have shown that the kinetics of Pb (II) adsorption onto other EPS extracted from various bacteria (e.g. *Paenibacillus polymyxa*, *Rhodococcus erythropolis*) also followed pseudo-second order kineticsmodels^17^,^18^. In accordance with the pseudo second-order kinetics mechanism, the Pb (II) uptake process could be due to chemisorptions^27^,^28^. The pseudo-second order rate equation of Pb (II) adsorption onto EPS was expressed as a function of temperature (295.15 K, 305.15 and 315.15 K) by the Arrhenius equation(2):


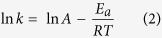
Here *Ea* is the Arrhenius activation energy (kJ mol^−1^), *A* is the Arrhenius factor, *R* is the ideal gas constant, *T* is the solution temperature (K) and *k* is the rate constant (*k2*) of the pseudo-second order rate model. To estimate the Arrhenius activation energy, ln(*k*) versus *1/T* was plotted in Fig. S2. The Arrhenius activation energy calculated from the slope was found to be 42.46 kJ mol^−1^. The physisorption process usually has energies in the range of 5–40 kJ mol^−1^ while the chemisorptions process has higher activation energy (40–800 kJ mol^−1^)^29^. This result confirmed that the nature of the adsorption process was chemisorption.

When the Pb (II) ion solution is mixed with the biosorbent, transport of the Pb (II) ions from the solution through the interface between the solution and the biosorbent and into the particle pores is effective^30^. Thus, predicting the rate-limiting step is another important factor to be considered in the sorption process. In the process of adsorption, there are essentially four consecutive stages by porous adsorbents^27^: 1) solute transfer from the bulk solution to the boundary film surrounding the particle, 2) diffusion from the boundary film to the exterior surface of the adsorbent, 3) solute diffusion from the surface into the pore of adsorbent and 4) solute sorption by physisorptions or chemisorptions. Providing sufficient agitation to avoid particle and solute gradients makes it possible to ignore the bulk diffusion. The last step is assumed to be rapid and considered to be negligible in the uptake of inorganic and organic compounds^30^. Thus, the intra-particle diffusion model and the liquid-film diffusion model were employed to distinguish between sorption controlled by intraparticle diffusion and film diffusion and the results were shown in Fig. 3(c,d)^31^,^32^. The plots *q*_*t*_ vs. *t*^0.5^ had lower regression coefficient of 0.76 at 295.15 K, 0.85 at 305.15 K and 0.86 at 315.15 K. Significantly, the plots did not have zero intercept (the intercept varies from 65 to 85). Thus, the plots did not fit intra-particle diffusion and it was not likely to be the controlling factor in kinetics process and large intercepts suggested that adsorption occurred mainly on the surface. The mechanism of the interactions might be governed by the slow transport of the metal ions from the liquid phase up to the biosorbent. Applying the liquid film diffusion model, it was observed that the plots were linear (*R*^*2*^: 0.87–0.92) with very small intercepts (0.6 to 2.1). Although the plots did not exactly pass through the origin, the small intercepts indicated that liquid film diffusion might have main role to play in the kinetics of biosorption of Pb (II) onto EPS.

The different kinetic models are important for indicating the possible mechanism of biosorption and identifying the rate-limiting step in the whole process. In the present case, the pseudo-second order model had the better fit with the experimental data. Therefore, it was likely that the nature of this process was chemisorption, and liquid-film diffusion might be the rate-limiting step in the kinetics of adsorption of Pb (II) onto EPS.

#### Thermodynamics studies

The values of thermodynamic parameters are relevant for the practical application of adsorption process. The dependence of temperature on adsorption of Pb (II) onto EPS of *Klebsiella* sp. J1 was evaluated using Vant Hoff’s equation. The variation in the extent of biosorption with respect to temperature had been explained based on thermodynamic parameters viz. Gibbs free energy change, enthalpy change and entropy change. The values of *ΔH*^*o*^ and *ΔS*^*o*^ were calculated from the intercept and slope of a plot of *ln k0* vs. *1/T*, shown in Fig. S2. The thermodynamic parameter values were given in Table 4. The Gibbs free energy of Pb (II) adsorption onto EPS *ΔG*^*o*^ was −9.72, −9.94, and −10.26 kJ/mol for the temperature of 295.15, 305.15 and 315.15 K, respectively. It was found that the negative *ΔG*^*o*^ at all temperatures confirming the adsorption process was spontaneous in nature and thermodynamically favorable. In addition, it was demonstrated in Fig. S3 that adsorption decreased with increasing temperature. The overall adsorption process seemed to be exothermic (*ΔH*^*o*^ = −1.76 kJ/mol). This result also supported the results shown in Table 3. Finally, the positive values of *ΔS*^*o*^ (0.03 kJ mol^−1^ K^−1^) was not very large and showed increased randomness at the solid/solution interface during the sorption of Pb (II) and good affinity of Pb (II) towards EPS. Some researchers have indicated that the positive values of *ΔS°* reflected some structural changed in the sorbent^33^,^34^.

#### Mechanism of Pb (II) biosorption

An effort was attempted to identify the major mechanism of adsorption of Pb (II) onto EPS of *Klebsiella* sp. J1. The zeta potential of Pb (II) solution and the adsorption capacity during the process were shown in Fig. S4. The adsorption capacity of Pb (II) first increased rapidly, then it increased steadily and reached adsorption equilibrium in 50 min. The zeta potential of Pb (II) solution was about 9.19 mV at pH 6. Although adding 200 mg L^−1^ electronegative EPS aroused a big change in the Zeta potential of system that it turned to be −4.15 mV rapidly, subsequent Zeta potential remained constant as adsorption capacity continued increasing. This result suggested that charge neutralization were not the main mechanisms of Pb (II) adsorption^17^.

The FT-IR spectra of unloaded and Pb (II) loaded forms of biosorbent EPS in the range of 400–4000 cm^−1^ were taken (Fig. S5) to identify the different functional groups in EPS that were responsible for Pb (II) sorption. The FT-IR spectrum of the original EPS showed several distinct and sharp absorptions at 3316 cm^−1^ (−OH or −NH_2_ groups), 2928 cm^−1^(C–H groups), 1653 cm^−1^ (C = O of protein bonds), 1535 cm^−1^ (N–H and C-N of amide groups), 1412 cm^−1^ (C = O of amide I) and at 1095 cm^−1^ (C-O-C and C-O of polysaccharide)^29^. The FT-IR spectra of EPS exposed to Pb (II) ions suggested no shifts or change in any of the characteristic absorbance bands with the exception of a peak shift at 3316 cm^−1^ and 1653 cm^−1^. This current results implied not only involvement of carboxyl groups in biosorption of Pb (II) ions, but also the possibility that biosorption could be taken place through an ion-exchange process rather than complexation.

**Figure 4:**
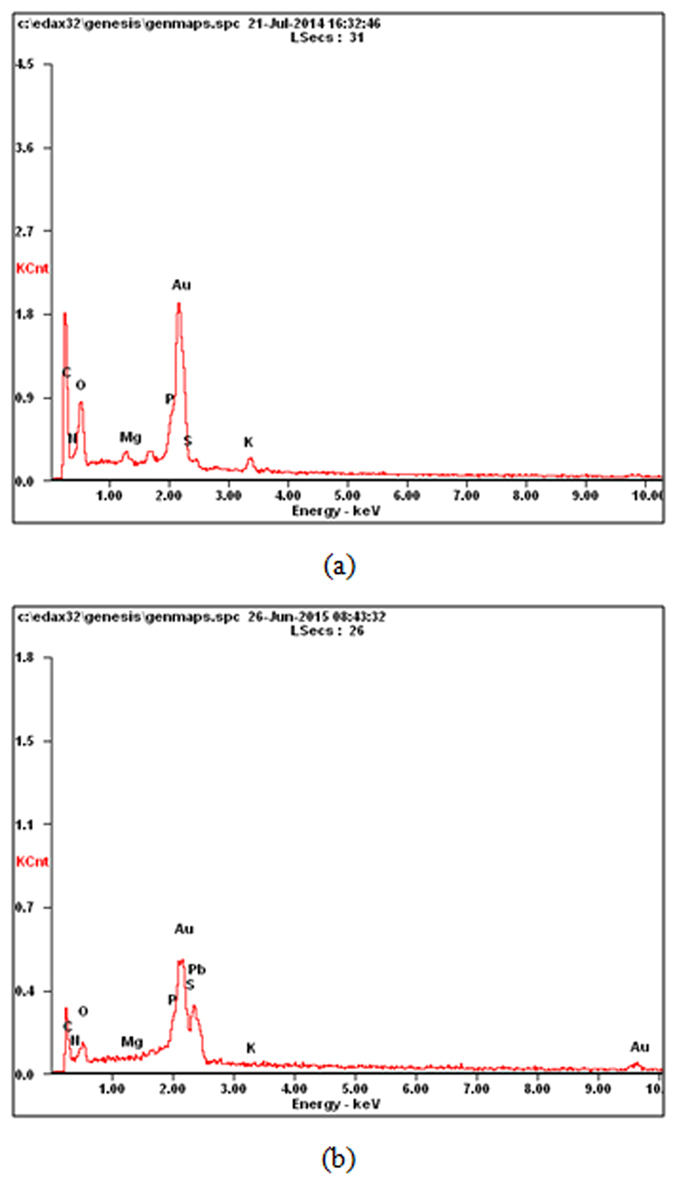
Typical EDAX spectra of EPS of *Klebsiella* sp. J1 before (**a**) and after (**b**) Pb (II) loaded.

Compared the typical EDAX spectra of the original EPS (Fig. 4(a)) with the Pb(II) loaded EPS (Fig. 4(b)), it was observed that the appearance of Pb (II) signal at about 2.2 keV and the disappearance of both K^+^ signal at about 3.4 keV and Mg^2+^ at about 1.2 keV after Pb (II) biosorption. These findings indicated ion-exchange mechanism played an important role in the biosorption process. For quantitative analysis in the search for further evidence on the major mechanism involved in the Pb (II) biosorption process, related element concentrations (K^+^, Mg^2+^ and Pb^2+^) in both the liquid and solid samples before and after Pb II) loading EPS were converted into molar equivalents as shown in Table 5. Positive and negative variations (±Δmequiv) in equivalents of related cations were also shown in order to infer changes in related cations involved during the mass transfer process occurring in both directions between the liquid and solid phases. Molar equivalents of both K^+^ and Mg^2+^ could be exchanged with Pb^2+^ molar equivalents in the process and the contribution rate of ion exchange to adsorption accounted for 85.72% (Δmequiv = −0.000541)^23^. Thus, according to the mass balance of elements participating in the biosorption process, ion-exchange was identified quantitatively as the major mechanism, which also be supported by the E-value obtained from Dubinin- Radushkevich isotherm.

In conclusion, laboratory-scale experiments suggested the EPS extracted from *Klebsiella* sp. J1 appeared as a potential and alternative biosorbent for the removal of Pb (II) from low concentrations aqueous medium. The maximum Pb (II) loading capacity of EPS was obtained as 99.5 mg g^−1^ at pH 6.0, which was better than the majority of other biosorbents, and the wastewater after its treatment was able to reach a level (0.1 mg L^−1^) that satisfied the EPA standard. The Langmuir and Dubinin-Radushkevich isotherms provided the better fit for the experimental data. Kinetic process was described best by a pseudo-second order model, while the liquid-film diffusion step might be the rate-limiting step. Based on both qualitative and quantitative analysis results, ion-exchange was identified as the major mechanism of adsorption Pb (II) by EPS. Molar equivalents of both K^+^ and Mg^2+^ could be exchanged with Pb^2+^ molar equivalents in the process and the contribution rate of ion exchange to adsorption accounted for 85.72% (Δmequiv = −0.000541).

## Methods

### Preparation and Characteristics of EPS

EPS were extracted from *Klebsiella* sp. J1 (CGMCC No. 6243), which was obtained from activated sludge in municipal wastewater treatment plants^35^.

*Klebsiella* sp. J1 cells were cultured and incubated in liquid medium using beef extract peptone medium at 30 °C for 24 h. Cells suspension of stock cultures was used as the seed medium. Four milliliter of inoculants was added into the 250 ml fermentation medium containing glucose 4.0 g, yeast extract 0.125 g, K_2_HPO_4_ 1.25 g, KH_2_PO_4_ 0.5 g, NaCl 0.025 g and MgSO_4_ 0.05 g. After cultivation at 30 °C for 24 h, centrifugation, ethanol extraction and dialysis, EPS dry powder was stored at 4 °C, and it would be dissolved in deionized water and utilized in the wastewater directly. EPS mainly composed of proteins, polysaccharides, a small quantity of nucleic acid and various metallic elements (e.g. Mg, K). Our previous research indicated the active constituents in EPS were proteins and polysaccharides, whose mass ratio was approximately 1.5, and proteins played a more important role compared with polysaccharides in the material’s activity^21^.

### Batch biosorption procedure

The stock Pb (II) solution (200 mg L^−1^) was prepared by dissolving lead salts (Pb(NO_3_)_2_) in deionized water. The desired working solutions were obtained by appropriate dilution of the stock solutions with deionized water.

Batch biosorption experiments were performed for different biosorbent dosages (20–320 mg L^−1^) or pH (1.0–6.0) at a room temperature of 20 ± 2 °C on a jar test apparatus (ZR4-6, Mingbo Environmental Technology Co. Ltd., Qingdao, China), containing 100 mL of 20 mg L^−1^ Pb (II) solution. A predetermined amount of EPS extracted from *Klebsiella* sp. J1 (20–320 mg L^−1^)was dosed to 100 mL of 20 mg L^−1^ Pb (II) solution with pH 6.0. Each water sample was rapidly mixed at 160 rpm for 1.0 min and then slowly mixed at 40 rpm for 2 min and settled for 120 min. After sorption, a supernatant sample was withdrawn for filtration using 0.45 μm cellulose acetate filter membrane, and free EPS as well as Pb (II)- EPS were remained on the filter membrane^18^. In order to avoid the error result from Pb (II) ions loaded filter membranedue, the control group without EPS was operated. The effect of pH on the biosorption capacity of EPS for Pb (II) was investigated. The pH of each metal solution was adjusted to the required value by adding 1 mol L^−1^ HNO_3_ or 1 mol L^−1^ of NaOH at the beginning of the experiment and not controlled afterwards. Inductively coupled plasma optical emission spectrometry (ICP-OES; Optima 5300 DV, PE, USA) was used to determine the concentration of metal ions in the aqueous solution, and the detection limit of ICP-OES was 10 ug L^−1^. The removal percentage of metal ions and the equilibrium uptake of EPS (*q*_*e*_) were calculated as follows:


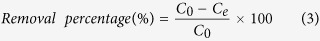



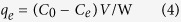


where *C*_*0*_ and *Ce* are the initial and equilibrium concentrations of metal solutions (mg L^−1^), *V* is the volume of the metal ions solution used (L), and *W* is the amount of EPS used (g)^2^. In this study, all experiments were performed in a batch setup with three replicates, and average values were reported. Standard deviations were found to be within ±1.3%. Error bars are not shown because of their smaller magnitude than that of the symbols used to plot the graphs.

### Equilibrium and Kinetic experiments

Isotherm studies were conducted at different initial Pb (II) concentrations (5 mg L^−1^ to 50 mg L^−1^) and different temperatures (295.15, 305.15 and 315.15 K). Langmuir, Freundlich and Dubinin-Radushkevich models were used to determine the adsorption equilibrium^10^. Kinetic data were obtained at designated time points (5 min to 70 min) and different temperatures of 295.15, 305.15 and 315.15 K. The adsorption kinetic data were analysed using pseudo-first and pseudo-second order kinetic models, the intra-particle diffusion and the liquid film diffusion model^2^. Thermodynamics parameters were evaluated using Vant Hoff’s equation at different temperatures of 295.15, 305.15 and 315.15 K^2^. The equations for these models and the methods of calculating the model parameters are presented in Table 6.

### Biosorption mechanism analysis

The zeta potential, Fourier transform infrared spectroscopy (FT-IR), energy dispersive X-ray analysis (EDAX) were employed to examine the interactions between EPS and the Pb (II) ions. The zeta potential of the system in whole process was measured with a Zetameter equipment (3000 Hsa, Malvern Instruments Co. Ltd., England). EPS samples (before and after Pb (II) loading) were prepared for FTIR and EDAX analysis. EPS loading Pb (II) samples under the optimal experimental conditions were collected, followed by rinse to remove free Pb (II). Then EPS samples (before and after Pb (II) loading) were processed by vacuum freeze-drying. The FT-IR spectra of EPS samples (before and after Pb (II) loading) in the range of 400–4000 cm^−1^ were recorded in a FTIR spectrometer (Nexus 870, Thermo Nicolet, USA) using the KBr disc technique. Energy dispersive X-ray analysis (EDAX) was employed to examine the changes of the elements on EPS samples (before and after Pb (II) loading).

For quantitative analysis in the search for further evidence on the mechanism involved in the Pb (II) biosorption process, related element concentrations in both the liquid and solid samples before and after Pb (II) biosorption on EPS were converted into molar equivalents according to Eq. (5).


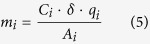


where *m*_*i*_ is the molar equivalents, *C*_*i*_ is the elemental concentrations (mg L^−1^), *δ* is the bulk biomass (g), *q*_*i*_ is the charge state of each cation, *A*_*i*_ is the relative atomic mass (mg mol^−1^)^23^. Positive and negative variations (±△mequiv) in equivalents of each cation were calculated in order to infer changes in all cations involved during the mass transfer process occurring in both directions between the liquid and solid phases.

## Additional Information

**How to cite this article**: Wei, W. *et al*. Biosorption of Pb (II) from aqueous solution by extracellular polymeric substances extracted from *Klebsiella* sp. J1: Adsorption behavior and mechanism assessment. *Sci. Rep.* **6**, 31575; doi: 10.1038/srep31575 (2016).

## Supplementary Material

Supplementary Information

## Figures and Tables

**Table 1:** Comparison of Pb (II) sorption capacity of EPS of *Klebsiella* sp.

Category	Biosorbent	pH	Temperature (°C)	Uptake capacity(mg g^−1^)	Ref.
Bacteria species	*Pistacia vera* L.	5.5	ambient temperature	43.4	36
	*Stenotrophomonas maltophilia*	7	ambient temperature	74.4	37
	*Saccharomyces cerevisiae*	6	25	116.7	25
	*Amanita rubescens*	5	20	38.4	24
waste biomaterials	Solanum melongena leaves	5	30	55.5	38
	Olive tree pruning	6	ambient temperature	23.3	8
	Dried aquatic plant	6	25	86.9	39
	Chestnut bur	5	ambient temperature	74.3	5
EPS	EPS of *A. fumigates*	5	25	20.1	40
	MBFR10543	6	ambient temperature	81.2	18
	EPS of *Colletotrichum* sp.	6	30	30.3	41
	EPS of *Klebsiella* sp. J1	6	ambient temperature	99.5	This work

J1 with other reported biosorbents under similar conditions.

**Table 2:** Langmuir, Freundlich and Dubinin-Radushkevich isotherm constants for the adsorption of Pb (II) onto EPS of *Klebsiella* sp. J1.

Model	Parameter	Tem. K		
		295.15	305.15	315.15
Langmuir	*q*_*m*_ (mg g^−1^)	153.85	149.25	147.06
	*b*(L mg^−1^)	0.05	0.05	0.05
	*R*_*L*_	0.27	0.28	0.29
	*R*^*2*^	0.92	0.92	0.92
Freundlich	*K*_*F*_ (mg g^−1^)	12.37	11.72	11.66
	*n*	1.68	1.67	1.66
	*R*^*2*^	0.77	0.78	0.78
Dubinin-Radushkevich	*q*_*m*_ (mg g^−1^)	100.36	95.99	93.24
	*E* (kJ mol^−1^)	8.22	8.57	8.98
	*R*^*2*^	0.93	0.91	0.91

**Table 3:** Kinetic parameters of pseudo-first order and pseudo-second order expressions for Pb (II) adsorption onto EPS of *Klebsiella* sp. J1 at different temperatures.

Tem. K	Pseudo-first order			Pseudo-second order		
	*k*_*1*_ (min^−1^)	*q*_*e*_ (mg g^−1^)	*R*^*2*^	*k*_*2*_ (min^−1^)×10^−2^	*q*_*e*_ (mg g^−1^)	*R*^*2*^
295.15	0.07	32.45	0.92	0.34	104.17	0.99
305.15	0.03	11.74	0.89	0.96	97.09	0.99
315.15	0.03	11.02	0.86	1.02	95.24	0.99

**Table 4:** Thermodynamic parameters for the adsorption of Pb (II) onto EPS of *Klebsiella* sp. J1.

Tem. K	***−ΔG*^*o*^** (kJ mol^−1^)	***ΔH*^*o*^** (kJ mol^−1^)	***ΔS*^*o*^** (kJ mol^−1^K^−1^)
295.15	9.72		
305.15	9.94	−1.76	0.03
315.15	10.26		

**Table 5:** Molar equivalent balance for related elements detected in liquid and solid phases before and after biosoption processes.

Related ions	Liquid phases			Solid Phases (EPS)		
	mequiv._before_	mequiv._after_	△mequiv.	mequiv._before_	mequiv._after_	△mequiv.
Pb^2+^	0.003810	0.000023	0.003787	0	0.003787	0.003787
K^+^	0	0.001831	0.001831	0.001831	0	−0.001831
Mg^2+^	0	0.001415	0.001415	0.001415	0	−0.001415
Total	0.003810	0.003269	−0.000541	0.003246	0.003787	0.000541

**Table 6:** The models and equations used for the adsorption of Pb (II) by EPS of *Klebsiella* sp. J1.

	Model	Equation	Model parameters
Adsorption isotherm	Langmuir	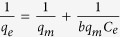	*q*_*e*_ = equilibrium capacity of Pb (II) adsorbed onto EPS (mg g^−1^), *C*_*e*_ = equilibrium concentration of Pb (II) solution (mg L^−1^), *q*_*m*_ = maximum adsorption capacity (mg g^−1^), and *b* = energy of adsorption (L mg^−1^);
	Freundlich	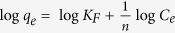	*q*_*e*_ = equilibrium capacity of Pb (II) adsorbed onto EPS (mg g^−1^), *C*_*e*_ = equilibrium concentration of Pb (II) solution (mg L^−1^), *K*_*F*_ = Freundlich constants (mg g^−1^), *n* = adsorption intensity of the adsorbent;
	Dubinin-Radushkevich	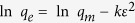	*q*_*e*_ = equilibrium capacity of Pb (II) adsorbed onto EPS (mg g^−1^), *q*_*m*_ = maximum adsorption capacity (mg g^−1^), *k* = energy of adsorption (mol^2^ kJ^−2^), *ε* is the Polanyi potential = *RT ln*(*1 + 1/Ce*), *R* = gas constant (kJ K^−1^ mol^−1^), *T* = temperature (K), *C*_*e*_ = equilibrium concentration of Pb (II) solution (mg L^−1^). *E* is the mean free energy of adsorption = (*2k*)^*−0.5*^ (kJ mol^−1^).
Adsorption kinetics	Pseudo-first order kinetic	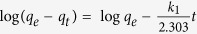	*q*_*e*_ = equilibrium adsorption capacity (mg g^−1^), *q*_*t*_ = adsorption (mg g^−1^) at any time t (h), *k*_*1*_ = first-order rate constant (h^−1^);
	Pseudo-second order kinetic	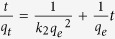	*q*_*e*_ = equilibrium adsorption capacity (mg g^−1^), *q*_*t*_ = adsorption (mg g^−1^) at any time t (h), *k*_*2*_ = the second-order rate constant (mg g^−1^ h^−1^);
	Intra-particle diffusion	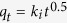	*q*_*t*_ = adsorption (mg g^−1^) at any time t (h), *k*_*i*_ = intra-particle diffusion rate constant (mg g^−1^ h^−0.5^);
	Liquid film diffusion	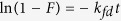	*F* = fractional attainment of adsorption equilibrium; *k*_*fd*_ = adsorption rate constant.
**Adsorption thermodynamics**	Vant Hoff’s equation	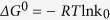 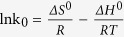	*ΔG*^*o*^ = Gibbs free energy change (kJ mol^−1^), *R* = the gas constant (J mol^−1^ K^−1^), *T* = temperature (K), *k0* = the equilibrium constant, *ΔH*^*o*^ = enthalpy change (kJ mol^−1^), *ΔS*^*o*^ = entropy change (kJ mol^−1^ K^−1^).

## References

[b1] Nguyen T. C. . Simultaneous adsorption of Cd, Cr, Cu, Pb, and Zn by an iron-coated Australian zeolite in batch and fixed-bed column studies. Chem. Eng. J. 270, 393–404 (2015).

[b2] Karatas M. Removal of Pb (II) from water by natural zeolitic tuff: kinetics and thermodynamics. J. Hazard. Mater. 199, 383–389 (2012). 22137175 10.1016/j.jhazmat.2011.11.035

[b3] Bingöl D., Hercan M., Elevli S. & Kılıç E. Comparison of the results of response surface methodology and artificial neural network for the biosorption of lead using black cumin. Bioresour. Technol. 112, 111–115 (2012). 22425399 10.1016/j.biortech.2012.02.084

[b4] Agelidis T., Fytianos K. & Vasilikiotis G. Kinetic study of lead cementation by iron powder in wastewater. Chemosphere. 14, 1001–1012 (1985).

[b5] Kim N., Park M. & Park D. A new efficient forest biowaste as biosorbent for removal of cationic heavy metals. Bioresour. Technol. 175, 629–632 (2015). 25467000 10.1016/j.biortech.2014.10.092

[b6] Gupta V. K. & Rastogi A. Equilibrium and kinetic modelling of cadmium(II) biosorption by nonliving algal biomass *Oedogonium* sp from aqueous phase. J. Hazard. Mater. 153, 759–766 (2008). 17942222 10.1016/j.jhazmat.2007.09.021

[b7] Sounthararajah D., Loganathan P., Kandasamy J. & Vigneswaran S. Adsorptive removal of heavy metals from water using sodium titanate nanofibres loaded onto GAC in fixed-bed columns. J. Hazard. Mater. 287, 306–316 (2015). 25668299 10.1016/j.jhazmat.2015.01.067

[b8] Ronda A., Della Zassa M., Martín-Lara M., Calero M. & Canu P. Combustion of a Pb (II)-loaded olive tree pruning used as biosorbent. J. Hazard. Mater. 308, 285–293 (2016). 26855182 10.1016/j.jhazmat.2016.01.045

[b9] Wang J. & Chen C. Biosorbents for heavy metals removal and their future. Biotechnol. Adv. 27, 195–226 (2009). 19103274 10.1016/j.biotechadv.2008.11.002

[b10] Cabuk A., Akar T., Tunali S. & Gedikli S. Biosorption of Pb(II) by industrial strain of *Saccharomyces cerevisiae* immobilized on the biomatrix of cone biomass of Pinus nigra: Equilibrium and mechanism analysis. Chem. Eng. J. 131, 293–300 (2007).

[b11] de Luna M. D. G., Flores E. D., Cenia M. C. B. & Lu M.-C. Removal of copper ions from aqueous solution by adlai shell (*Coix lacryma-jobi* L.) adsorbents. Bioresour. Technol. 192, 841–844 (2015). 26081160 10.1016/j.biortech.2015.06.018

[b12] Hadi P., Barford J. & McKay G. Toxic Heavy Metal Capture Using a Novel Electronic Waste-Based Material Mechanism, Modeling and Comparison. Environ. Sci. Technol. 47, 8248–8255 (2013). 23837660 10.1021/es4001664

[b13] d’Abzac P. . Characterization of the mineral fraction associated to extracellular polymeric substances (EPS) in anaerobic granular sludges. Environ. Sci. Technol. 44, 412–418 (2009). 19950914 10.1021/es901912g

[b14] Sheng G., Yu H. & Li X. Extracellular polymeric substances (EPS) of microbial aggregates in biological wastewater treatment systems: a review. Biotechnol. Adv. 28, 882–894 (2010). 20705128 10.1016/j.biotechadv.2010.08.001

[b15] Wang J. . Competitive adsorption of heavy metal by extracellular polymeric substances (EPS) extracted from sulfate reducing bacteria. Bioresour. Technol. 163, 374–376 (2014). 24841491 10.1016/j.biortech.2014.04.073

[b16] Cao B. . Contribution of extracellular polymeric substances from *Shewanella* sp. HRCR-1 biofilms to U (VI) immobilization. Environ. Sci. Technol. 45, 5483–5490 (2011). 21627155 10.1021/es200095j

[b17] Feng J. . The adsorption behavior and mechanism investigation of Pb (II) removal by flocculation using microbial flocculant GA1. Bioresour. Technol. 148, 414–421 (2013). 24077150 10.1016/j.biortech.2013.09.011

[b18] Guo J. & Yu J. Sorption characteristics and mechanisms of Pb (II) from aqueous solution by using bioflocculant MBFR10543. Appl. microbiol. biotechnol. 98, 6431–6441 (2014). 24816617 10.1007/s00253-014-5681-z

[b19] Lin J. & Harichund C. Isolation and characterization of heavy metal removing bacterial bioflocculants. Afr. J. Microbiol. Res. 5, 599–607 (2011).

[b20] Noghabi K. A., Zahiri H. S. & Yoon S. C. The production of a cold-induced extracellular biopolymer by *Pseudomonas fluorescens* BM07 under various growth conditions and its role in heavy metals absorption. Process Biochem. 42, 847–855 (2007).

[b21] Wei W. . Synergetic effects and flocculation behavior of anionic polyacrylamide and extracellular polymeric substrates extracted from *Klebsiella* sp. J1 on improving soluble cadmium removal. Bioresour. Technol. 175, 34–41 (2015). 25459801 10.1016/j.biortech.2014.10.044

[b22] Yang J. . Competitive adsorption of heavy metals by extracellular polymeric substances extracted from *Klebsiella* sp. J1. Bioresour. Technol. 196, 533–539 (2015). 26291413 10.1016/j.biortech.2015.08.011

[b23] Módenes A., Espinoza-Quiñones F., Santos G., Borba C. & Rizzutto M. Assessment of metal sorption mechanisms by aquatic macrophytes using PIXE analysis. J. Hazard. Mater. 261, 148–154 (2013). 23921177 10.1016/j.jhazmat.2013.07.020

[b24] Sarı A., Tuzen M., Cıtak D. & Soylak M. Adsorption characteristics of Cu (II) and Pb (II) onto expanded perlite from aqueous solution. J. Hazard. Mater. 148, 387–394 (2007). 17386972 10.1016/j.jhazmat.2007.02.052

[b25] Ma X. . Efficient biosorption of lead (II) and cadmium (II) ions from aqueous solutions by functionalized cell with intracellular CaCO_3_ mineral scaffolds. Bioresour. Technol. 185, 70–78 (2015). 25755015 10.1016/j.biortech.2015.02.074

[b26] Ghorai S., Sinhamahpatra A., Sarkar A., Panda A. B. & Pal S. Novel biodegradable nanocomposite based on XG-g-PAM/SiO_2_: application of an efficient adsorbent for Pb^2+^ ions from aqueous solution. Bioresour. Technol. 119, 181–190 (2012). 22728199 10.1016/j.biortech.2012.05.063

[b27] Dundar M., Nuhoglu C. & Nuhoglu Y. Biosorption of Cu (II) ions onto the litter of natural trembling poplar forest. J. Hazard. Mater. 151, 86–95 (2008). 17601663 10.1016/j.jhazmat.2007.05.055

[b28] Vadivelan V. & Kumar K. Equilibrium, kinetics, mechanism, and process design for the sorption of methylene blue onto rice husk. J. Colloid. Interf. Sci. 286, 90–100 (2005). 10.1016/j.jcis.2005.01.007 15848406

[b29] Chakravarty S. . Removal of Pb (II) ions from aqueous solution by adsorption using bael leaves (*Aegle marmelos*). J. Hazard. Mater. 173, 502–509 (2010). 19765896 10.1016/j.jhazmat.2009.08.113

[b30] Djeribi R. & Hamdaoui O. Sorption of copper (II) from aqueous solutions by cedar sawdust and crushed brick. Desalination 225, 95–112 (2008).

[b31] Weber W. & Morris J. Kinetics of adsorption on carbon from solution. J. Sanit. Eng. Div. Am. Soc. Civ. Eng. 89, 31–60 (1963).

[b32] Boyd G., Adamson A. & Myers Jr L. The exchange adsorption of ions from aqueous solutions by organic zeolites. II. Kinetics1. J. Am. Chem. Soc. 69, 2836–2848 (1947). 20270838 10.1021/ja01203a066

[b33] Ho Y.-S. Removal of copper ions from aqueous solution by tree fern. Water Res. 37, 2323–2330 (2003). 12727241 10.1016/S0043-1354(03)00002-2

[b34] Manohar D., Krishnan K. A. & Anirudhan T. Removal of mercury (II) from aqueous solutions and chlor-alkali industry wastewater using 2-mercaptobenzimida zole-clay. Water Res. 36, 1609–1619 (2002). 11996349 10.1016/s0043-1354(01)00362-1

[b35] Xing J. . Removal efficiency and mechanism of sulfamethoxazole in aqueous solution by bioflocculant MFX. J. Anal. Methods Chem. 2013, 1–8 (2013). 10.1155/2013/568614 PMC3588202 23476893

[b36] Yetilmezsoy K., Demirel S. & Vanderbei R. J. Response surface modeling of Pb (II) removal from aqueous solution by *Pistacia vera* L.: Box–Behnken experimental design. J. Hazard. Mater. 171, 551–562 (2009). 19577844 10.1016/j.jhazmat.2009.06.035

[b37] Sen S. K., Raut S., Dora T. K. & Mohapatra P. K. D. Contribution of hot spring bacterial consortium in cadmium and lead bioremediation through quadratic programming model. J. Hazard. Mater. 265, 47–60 (2014). 24333714 10.1016/j.jhazmat.2013.11.036

[b38] Yuvaraja G., Krishnaiah N., Subbaiah M. V. & Krishnaiah A. Biosorption of Pb (II) from aqueous solution by *Solanum melongena* leaf powder as a low-cost biosorbent prepared from agricultural waste. Colloid Surfaces B. 114, 75–81 (2014). 10.1016/j.colsurfb.2013.09.039 24176885

[b39] Tang Y., Chen L., Wei X., Yao Q. & Li T. Removal of lead ions from aqueous solution by the dried aquatic plant, *Lemna perpusilla* Torr. J. Hazard. Mater. 244, 603–612 (2013). 23182246 10.1016/j.jhazmat.2012.10.047

[b40] Li W.-W. & Yu H.-Q. Insight into the roles of microbial extracellular polymer substances in metal biosorption. Bioresour. Technol. 160, 15–23 (2014). 24345430 10.1016/j.biortech.2013.11.074

[b41] da Silva L. J., de Rezende Pinto F., do Amaral L. A. & Garcia-Cruz C. H. Biosorption of cadmium (II) and lead (II) from aqueous solution using exopolysaccharide and biomass produced by *Colletotrichum* sp. *Desalin*. Water Treat. 52, 7878–7886 (2014).

