# Supplementary material for: Biosorption of Pb (II) from aqueous solution by extracellular polymeric substances extracted from Klebsiella sp. J1: Adsorption behavior and mechanism assessment
Source: Sci Rep. 2016 Aug 12;6:31575. doi: 10.1038/srep31575 (PMC4981841; doi:10.1038/srep31575)
Supplement: Supplementary Information [file srep31575-s1.pdf]

Supplementary Information

Biosorption of Pb (II) from aqueous solution by extracellular polymeric substances extracted from *Klebsiella* sp. J1: Adsorption behavior and mechanism assessment

Wei Wei[1,2], Qilin Wang[3], Ang Li[1,2,*], Jixian Yang[1,2,*], Fang Ma[1,2], Shanshan Pi[1,2], Dan Wu[1,2]

[1]School of Municipal and Environmental Engineering, Harbin Institute of Technology, Harbin 150090, People's Republic of China;

[2]State Key Laboratory of Urban Water Resource and Environment, Harbin Institute of Technology, Harbin 150090, People's Republic of China

[3]Advanced Water Management Centre, The University of Queensland, St Lucia, Queensland 4072, Australia

Correspondence and requests for materials should be addressed to

A.L. (email: ang.li.harbin@gmail.com) and

J.Y. (email: yangxj@hit.edu.cn).

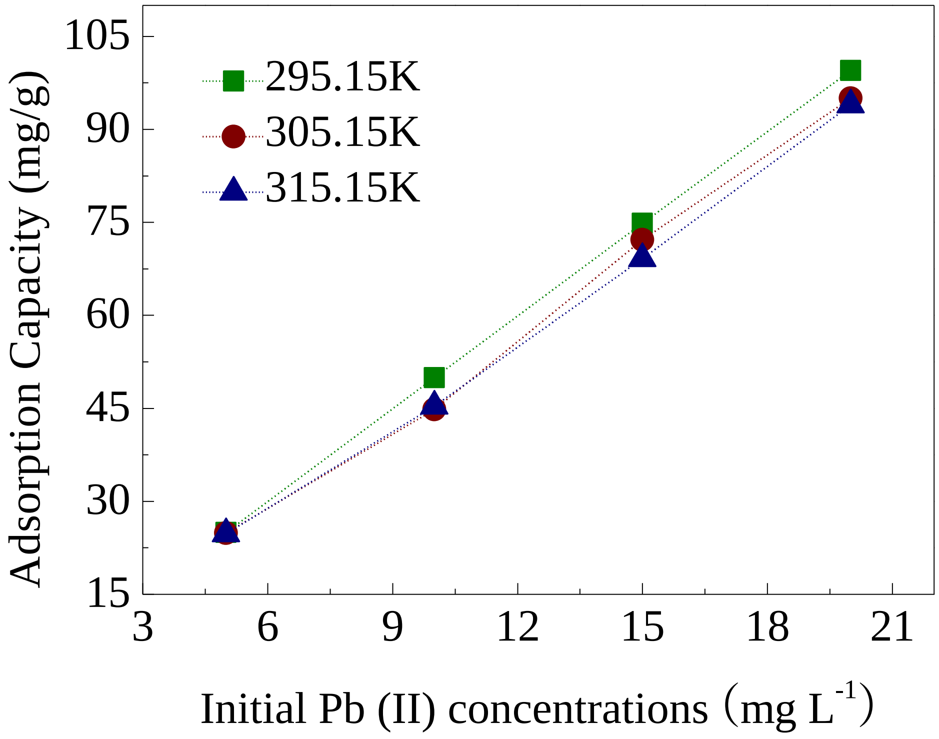

Figure S1. Effect of lower initial metal concentration for biosorption of Pb (II).

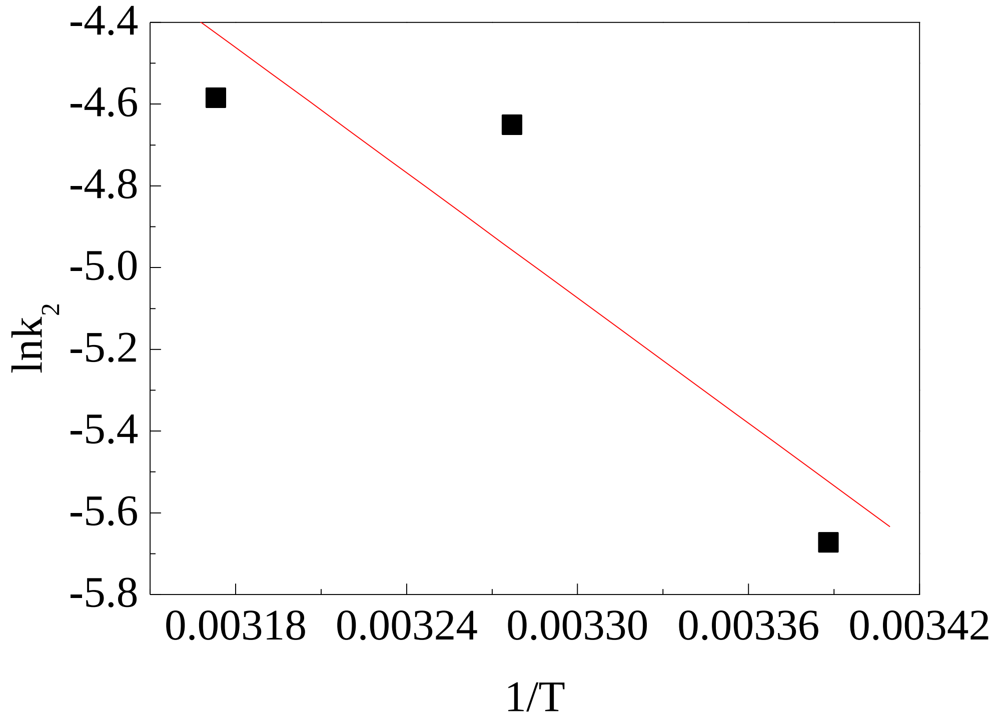

Figure S2. Arrhenius equation plots for biosorption of Pb (II) onto EPS of *Klebsiella* sp. J1.

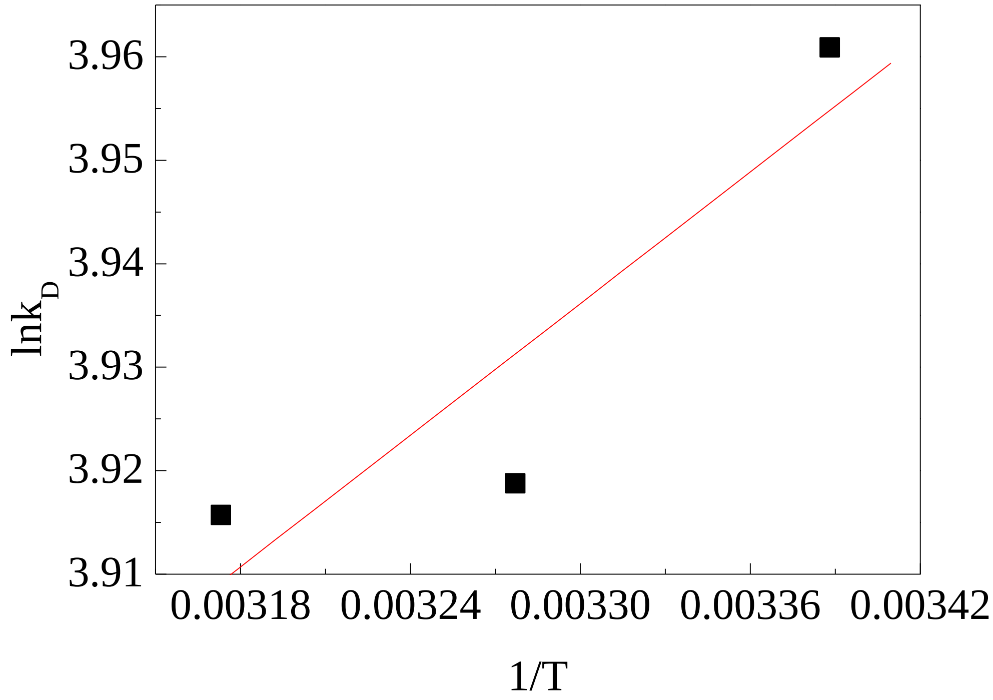

Figure S3. Van’t Hoff plots for estimation of thermodynamic parameters.

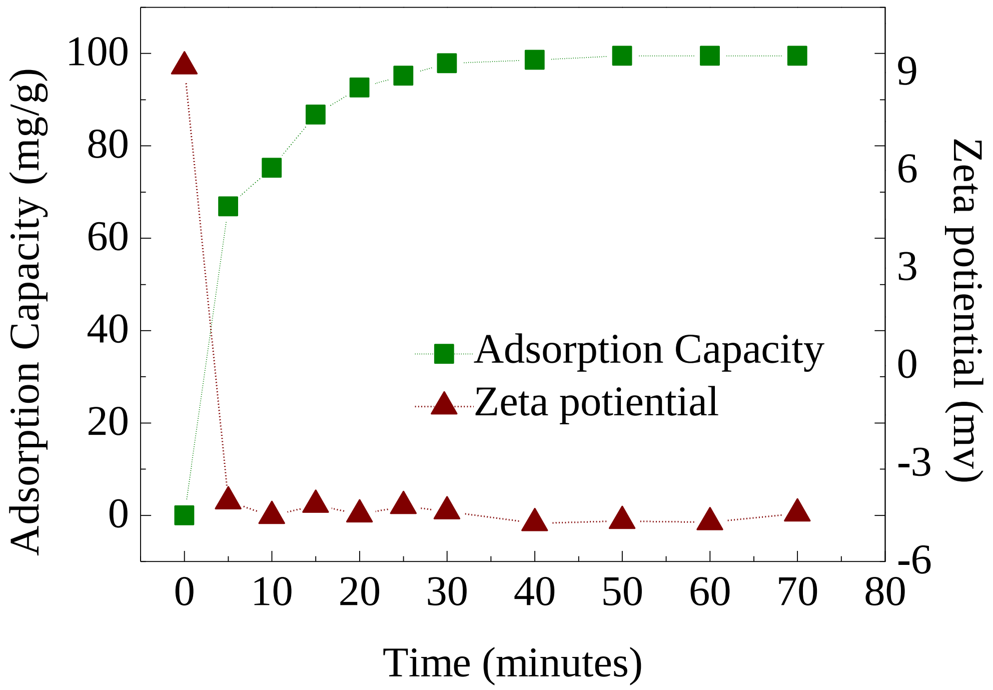

Figure S4. Zeta potential and adsorption capacity of EPS of *Klebsiella* sp. J1 for Pb (II) during the whole process.

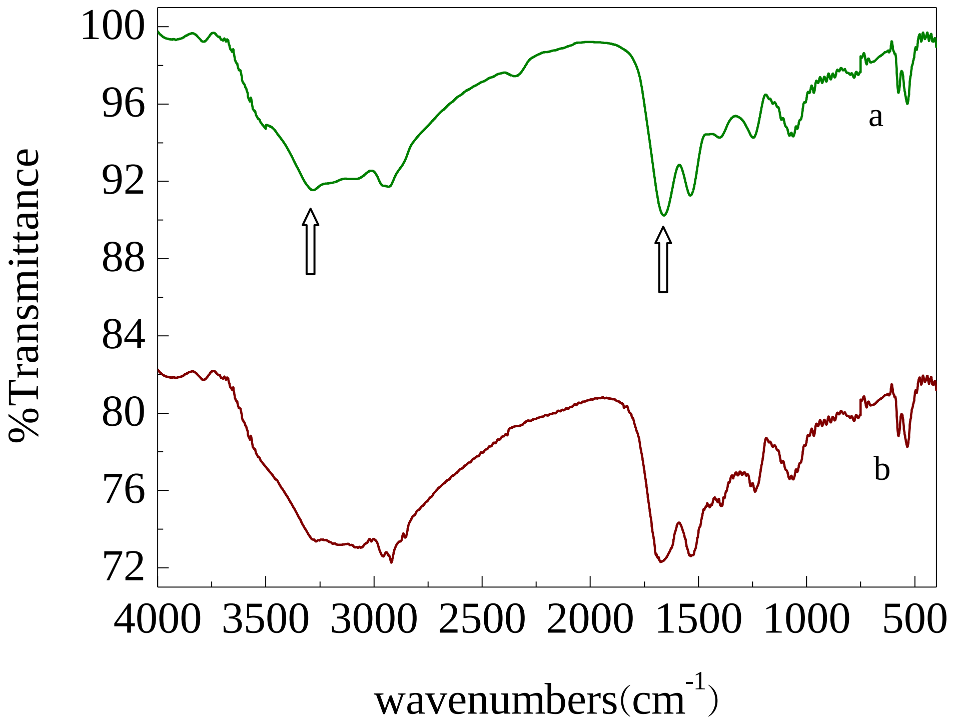

Figure S5. FTIR spectra of EPS of *Klebsiella* sp. J1 before (a) and after (b) Pb (II) loaded
